# Differences in Susceptibility to Okadaic Acid, a Diarrhetic Shellfish Poisoning Toxin, between Male and Female Mice

**DOI:** 10.3390/toxins5010009

**Published:** 2012-12-27

**Authors:** Hodaka Suzuki

**Affiliations:** Division of Biomedical Food Research, National Institute of Health Sciences, 1-18-1 Kamiyoga, Setagaya-ku, Tokyo 158-8501, Japan; E-Mail: hodaka@nihs.go.jp; Tel.: +81-3-3700-1141 ext.536; Fax: +81-3-3700-9527

**Keywords:** sex difference, okadaic acid, mouse bioassay, diarrhetic shellfish poisoning (DSP) toxin

## Abstract

The mouse bioassay (MBA) for diarrhetic shellfish poisoning (DSP) toxins has been widely used in many countries of the world. In the Japanese and EU methods, male mice are designated to be used for MBA. Female mice were described to be less susceptible than male mice. To the best of our knowledge, however, there have been no reports on the details of sex differences in susceptibility to DSP toxins. In this study, we investigated whether, and to what extent, female mice are less sensitive to DSP toxins. A lethal dose of okadaic acid (OA), one of the representative DSP toxins, was injected intraperitoneally into mice. The mice were observed until 24 hours after injection. Both male and female mice of ICR and ddY strains, which are designated in the Japanese official method, were compared. All the mice were four weeks old and weighed 18–20 g. The experiments were repeated twice. The lethality was 70%–100%. Survival analysis showed no sex differences in susceptibility to OA, but ICR female mice showed significant resistance compared with other groups in one out of two trials. These results indicate that sex differences were not clear but, nonetheless, male mice showed more stable results.

## 1. Introduction

Diarrhetic shellfish poisoning (DSP) is a toxic syndrome, resulting from the consumption of shellfish contaminated with algal toxins produced by marine dinoflagellates [1]. The most common symptoms of the illness are diarrhea, nausea, and vomiting. DSP has been recognized as a worldwide problem in public health since the first report of its occurrence in Japan by Yasumoto *et* *al*. [2].

The mouse bioassay (MBA) was first determined as the official method for DSP toxin detection in Japan in 1981 [3]. Since then, the MBA for DSP toxins, also known as Yasumoto’s method, has been widely used in many countries of the world [4], with some modifications, although the alternative chemical methods such as HPLC and LC-MS, and/or biochemical methods such as ELISA and phosphatase-inhibition assay for detecting DSP toxins have been developed recently [4]. In the Japanese official MBA method [3], the extract from the shellfish sample is injected intraperitoneally (i.p.) into three male ddY or ICR strain mice weighing 16–20 g. The sample is deemed positive (contaminated) if at least two of the three mice die within 24 hours of the injection. The EU-harmonized standard operating procedure for determination of lipophilic toxins by mouse bioassay [5] states “Use male albino mice Swiss strain (CD1 is recommended) weighing between 17 g and 22 g.” As mentioned above, only male mice are used for the bioassay in both protocols in Japan and EU. Yasumoto, who established the basis of the MBA for DSP toxins, clearly noted that female mice were less sensitive compared to male mice and should not be used for the assay, but no details or references were given in the paper [3]. On the other hand, reported lethal dose 50 (LD50) values by i.p. injection of okadaic acid (OA), one of the representative components of DSP toxins, were almost comparable between male and female mice (male 4 μg/~20 g mouse [6], female 192~204 μg/kg [7]).

In this study, we aimed to examine whether, and to what extent, female mice are less susceptible to DSP toxins compared with male mice.

## 2. Results and Discussion

### 2.1. Lethality after OA Inoculation

The lethality after OA inoculation was 70%–100% at 24 hours after injection in both experiments (Table 1 and Table 2). Fisher’s exact test showed no significant differences (*p* < 0.05) in the lethality among the groups in both experiments.

### 2.2. Survival Analysis of the Mice after OA Inoculation

**Figure 1:**
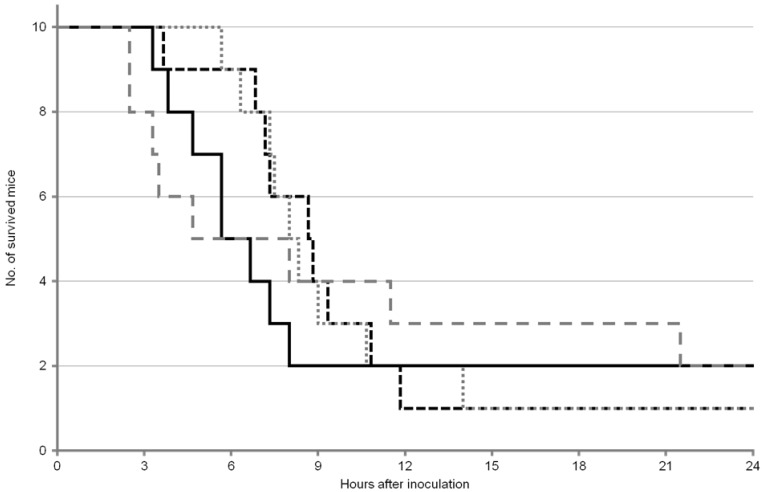
Survival curves of mice after OA inoculation in the first experiment. ICR male: 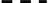; ICR female: 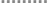; ddY male: 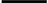; ddY female: 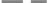.

**Figure 2:**
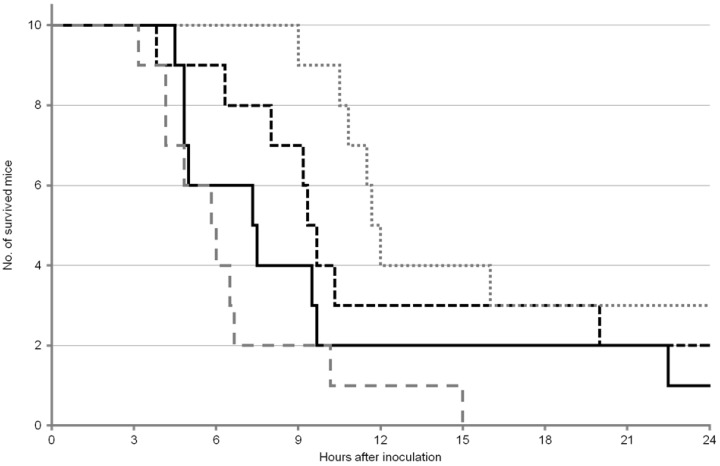
Survival curves of mice after OA inoculation in the second experiment. ICR male: 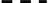; ICR female: 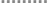; ddY male: 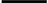; ddY female: 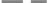.

The survival curves of the mice after OA inoculation are shown in Figure 1 and Figure 2. For survival analysis, both log-rank test and Gehan–Wilcoxon test were used. It is generally considered that the log-rank test is more standard, and the Gehan–Wilcoxon test gives more weight to deaths at earlier time points. In the first experiment, no statistically significant differences were found among any groups of mice (Table 3), although the median survival time was slightly longer in ICR male and female mice compared with ddY male and female mice (Table 1). On the other hand, in the second experiment, statistically significant difference was found between ICR female mice and ddY female mice (Table 4), but not among other groups. The median survival time of ICR female mice was twice as long as that of ddY female mice (Table 4).

### 2.3. Comparison of the Results of the First and Second Experiments

The results of each sex and strain of mice were statistically compared between the first and second experiments. Significant differences were found only between the first and second experiments of ICR female mice (Table 5).

**Table 1:** The lethality and median survival time after okadaic acid (OA) inoculation in the first experiment.

Strain	lethality	survived	dead	median survival time (h)
ICR male	90%	1	9	8.75
ICR female	90%	1	9	8.17
ddY male	80%	2	8	6.17
ddY female	80%	2	8	6.33

Fisher's exact test: *p* = 1.000 (not significant).

**Table 2:** The lethality and median survival time after OA inoculation in the second experiment.

Strain	lethality	survived	dead	median survival time (h)
ICR male	80%	2	8	9.50
ICR female	70%	3	7	11.83
ddY male	90%	1	9	7.42
ddY female	100%	0	10	5.92

Fisher's exact test: *p* = 0.463 (not significant).

**Table 3:** The survival analysis of the mice after OA inoculation in the first experiment.

Strain		Strain	Log-Rank test	Gehan-Wilcoxon test
among all strains			NS	NS
ICR male	*vs*	ICR female	NS	NS
ddY male	*vs*	ddY female	NS	NS
ICR male	*vs*	ddY male	NS	NS
ICR female	*vs*	ddY female	NS	NS

NS: not significant.

**Table 4:** The survival analysis of the mice after OA inoculation in the second experiment.

Strain		Strain	Log-Rank test	Gehan-Wilcoxon test
among all strains			***	***
ICR male	*vs*	ICR female	NS	NS
ddY male	*vs*	ddY female	NS	NS
ICR male	*vs*	ddY male	NS	NS
ICR female	*vs*	ddY female	***	***

***: *p* < 0.001, NS: not significant.

**Table 5:** The survival analysis of each strain of mice after OA inoculation between the first and second experiments.

Strain	Log-Rank test	Gehan-Wilcoxon test
ICR male	NS	NS
ddY male	NS	NS
ICR female	*	**
ddY female	NS	NS

*: *p* < 0.05, **: *p* < 0.01, NS: not significant

## 3. Experimental Section

### 3.1. Animals

Specific-pathogen-free, 4 weeks old, male and female mice of strains ICR and ddY were purchased from Japan SLC Inc. (Shizuoka, Japan). These 2 strains are designated to be used in the Japanese official MBA method. The mice were adapted to our animal facility for 1 day and used at 18–20 g body weight. The room lighting was 12 hours light (09:00~21:00)–12 hours dark (21:00~09:00) cycle. The mice were housed in plastic cages with wood chip bedding and commercial pellets (CRF-1; Charles River Japan Inc., Kanagawa, Japan) and tap water were provided *ad* *libitum*. All animal experiments were conducted with the approval of the Animal Care and Use Committee of the National Institute of Health Sciences, Japan.

The ddY strain is a closed-colony outbred mouse strain and widely used in Japan, but not in other countries of the world. This strain was first established as the dd strain, probably German Swiss albino mice origin, at the Institute of Infectious Diseases of Tokyo University (Denken) in 1943. Then, dd mice were provided to, maintained and finally subdivided as ddY mice at the National Institute of Health (Yoken) in 1953. The strain name, ddY, stands for the capital letters of Deutschland (Germany), Denken, and Yoken [8].

### 3.2. Chemicals

OA was purchased from LC Laboratories (Woburn, MA, U.S.A.). OA inocula were prepared as described previously [9]. Briefly, OA was first dissolved in acetone and then mixed with soybean oil. Soybean oil was used as a non-toxic vehicle for the lipophilic toxin. Acetone was removed by evaporation and the residue was suspended in 1% Tween 60 saline and sonicated. The final inocula contained 10% soybean oil. An inoculum prepared in one tube and mixed well was used in each experiment.

### 3.3. Experiment Design

A lethal dose of OA (4 μg/mL/mouse) [6,10] was injected i.p. into each mouse. Each group consisted of 10 mice. The mice were observed every 10 minutes until 12 hours after injection, and then every 30 minutes until 24 hours after injection. During the dark period, red lights were used for observation. No negative (vehicle) control (injected only 1% Tween 60 saline contained 10% soybean oil) was examined in this experiment, but no deaths of the negative control group were observed in our other experiments. The experiments were repeated twice. The experiment design basically followed the official MBA method for DSP toxins of Japan (and the EU). Humane endpoints were not applied in these experiments, because humane endpoints are not provided in the official methods.

### 3.4. Statistics

The lethality was statistically compared among the groups using Fisher’s exact test. Survival analysis was conducted using log-rank test and Wilcoxon–Gehan test. All statistical analyses were performed using the statistical package R version 2.13.0 [11].

## 4. Conclusions

In our two trials, sex differences were not found in susceptibility to OA. Significant differences were found only among the female mice groups, that is, between ICR female mice and ddY female mice in the second experiment, and between ICR female mice in the first and second experiments. Especially, ICR female mice in the second experiment showed significant resistance to OA compared with other groups.

The results of this study indicate that it might not be true that female mice are less sensitive than male mice and both male and female mice could be used for MBA of DSP, as AOAC MBA for paralytic shellfish poisoning (AOAC Official Method 959.08) [12]. However, it may be reasonable to use only male mice for obtaining more stable and reliable results in the MBA for DSP.
